# Editorial: Surgery and management of pituitary region tumours and their endocrine outcomes

**DOI:** 10.3389/fendo.2026.1794058

**Published:** 2026-02-06

**Authors:** Chandrasekaran Kaliaperumal

**Affiliations:** Department of Neurosurgery, Royal Infirmary of Edinburgh, Edinburgh, United Kingdom

**Keywords:** MRI brain, multi disciplinary approach, pituitary tumor, radiomics, WHO classification

Pituitary tumour management has undergone a remarkable transformation over the past two decades, driven by advances in diagnosis, treatment and systems of care worldwide. This evolution has allowed clinicians to offer safer, more effective and increasingly equitable treatment, while still needing to balance innovation with wisdom, common sense and the enduring foundations of medicine.

The World Health Organization (WHO) re-classification of pituitary tumours has placed greater emphasis on proliferative indices, lineage-specific transcription factors and molecular markers, refining prognosis and guiding adjuvant therapy decisions (1). This shift from purely morphological to integrated diagnoses has helped clinicians better stratify risk, anticipate aggressiveness and personalise follow-up strategies across different healthcare settings. At the same time, the biology of pituitary tumours continues to be explored beyond conventional pharmacology, including interest in nutraceuticals such as curcumin, whose antiproliferative and metabolic effects on tumour cells are being investigated in preclinical and translational studies in brain and other tumours, albeit not yet incorporated into standard of care for pituitary disease (2).

Neuroimaging has travelled a long road from invasive pneumoencephalography and lumbar puncture–induced pneumocephalus to high-resolution magnetic resonance imaging (MRI) with multiplanar and contrast-enhanced protocols for detailed sellar and parasellar assessment. Modern MRI does not simply localise a lesion; it characterises size, invasiveness, cavernous sinus and optic chiasm involvement, and increasingly supports pre-operative planning and risk assessment for both adenomas and more aggressive entities. Emerging MRI-based radiomics and texture analysis promise non-invasive insights into tumour consistency, subtype and behaviour, with early studies suggesting correlations with histological features and proliferative markers, although methodological heterogeneity and the need for external validation currently limit routine clinical adoption (3).

Surgically, endoscopic transsphenoidal approaches have become predominant in many centres, offering panoramic visualisation, angled views and improved access to complex or extended lesions, while microscopic transsphenoidal surgery remains a valuable technique in experienced hands. The modern pituitary neurosurgeon is therefore expected to be fluent in both endoscopic and microscopic approaches, selecting an approach that reflects tumour anatomy, patient factors and institutional expertise rather than technology alone. Artificial intelligence and digital tools now support teaching and training in skull base anatomy, approach planning and intra-operative decision-making, from virtual reality simulations to AI-assisted video analysis, potentially shortening learning curves but also underscoring the timeless warning that tools cannot replace judgment (3).

Clinically, the protean manifestations of pituitary tumours—from subtle, non-specific complaints to florid syndromes of hormone excess—continue to challenge timely diagnosis and comprehensive long-term care. Persistent endocrine morbidity, metabolic and cardiovascular risk, and quality-of-life impairment mean that “cure” extends far beyond radiological resection or biochemical normalisation. Visual function retains a central place in decision-making, with Optical Coherence Tomography (OCT) adding sensitive structural assessment of retinal nerve fibre and ganglion cell layers to standard perimetry, enabling detection of early or subclinical compromise in patients with chiasmal compression and informing the timing and urgency of intervention.

Multidisciplinary team (MDT) management has rightly become the standard of care, uniting endocrinologists, neurosurgeons, neuroradiologists, ophthalmologists, oncologists, pathologists and specialist nurses—often in “one-stop” pituitary clinics. This collaborative model helps optimise the sequencing of surgery, medical therapy and radiotherapy, reduces unwarranted variation in practice and promotes more efficient use of constrained healthcare resources, an increasingly important consideration in both high- and low-middle-income settings. Nonetheless, there remains a global responsibility to extend these principles beyond well-resourced centres, adapting evidence-based protocols to local infrastructure so that safe and affordable pituitary care does not become a privilege of geography (2).

Excellence in pituitary tumour management ultimately rests on a blend of expertise in both benign and malignant pathologies, judicious use of an ever-expanding technological armamentarium and unwavering reliance on clinical acumen and common sense.

Dr Harvey Cushing’s (Father of modern neurosurgery) quote that ‘*I would like to see the day when somebody would be appointed surgeon somewhere who had no hands, for the operative part is the least part of the work’* and Sir Robert Hutchison’s plea to to use ‘wisdom before knowledge’, ‘art before science’ and ‘common sense before cleverness’ remain strikingly relevant in an era of AI, radiomics and molecular profiling, challenging clinicians to ensure that wisdom continues to conduct the expanding global orchestra of pituitary care (4).
